# The health impacts of climate-related migration

**DOI:** 10.1186/s12916-017-0981-7

**Published:** 2017-12-11

**Authors:** Patricia Schwerdtle, Kathryn Bowen, Celia McMichael

**Affiliations:** 1 Nursing and Midwifery, Faculty of Medicine, Nursing and Health Sciences, Monash University, Peninsula Campus, McMahons Road, Frankston, VIC 3199 Australia; 2 Research School of Population Health, Australian National University, 52 Collins Street, Melbourne, VIC 3000 Australia; 3 The School of Geography, The University of Melbourne, Parkville, VIC 3010 Australia; 4 Médecins Sans Frontières, Glebe, Australia; 5 Institute of Public Health, Faculty of Medicine, Heidelberg University, Heidelberg, Germany; 6 Honorary Senior Fellow, Clean Air and Urban Landscapes Hub, University of Melbourne, Melbourne, Australia

**Keywords:** Climate change, Migration, Displacement, Relocation, Public health, Governance, Adaptation, Human mobility, Environmental change

## Abstract

**Background:**

Changes in climate, in conjunction with other drivers of mobility, shape human migration. While there is an increasing focus on the adaptive potential of migration, the health impacts of climate-related migration, including planned relocation and forced displacement, have not been thoroughly examined. The Intergovernmental Panel on Climate Change stated that migration is currently, and will increasingly be, influenced by environmental degradation and climate change, and that it needs to be addressed in a focused and coordinated manner.

**Discussion:**

This paper examines the links between climate change, migration, and health, considering diverse migration responses, including immobility, forced displacement and planned migration, as well as the associated health risks and opportunities in different contexts. Using case studies, the paper illustrates strategies to reduce the health risks associated with climate change-related migration.

**Conclusion:**

While there is an increasing body of research examining the climate change–migration nexus, a dual approach is now required. This approach must include debate and further research regarding the health consequences and responses associated with climate migration as well as immediate strengthening of health systems to make them both climate resilient and migrant inclusive.

## The nexus between climate change, migration, and health

Human migration in response to ecological change has been occurring since the origin of our species [1], yet the push that anthropogenic climate change is currently exerting on human migration is relatively new and gradually intensifying [2]. Environmental changes associated with increasing greenhouse gas concentrations include flooding, drought, increased frequency and intensity of climate-related disasters, and sea-level rise [3]. Globally, these environmental changes are shaping human migration, particularly through their intersection with other economic, political and social drivers of mobility [4]. Climate change acts as a threat multiplier, exacerbating existing sociopolitical and economic vulnerabilities, undermining livelihoods [5], inflating the risk of conflict [6], and making it difficult for people to remain in situ [7].

Moving beyond assumptions that climate change will lead to mass international displacement and threaten geopolitical security [8, 9], there is an increasingly nuanced understanding that human migration represents an adaptive response to the impacts of climate change [4, 10]. Indeed, there have been explicit efforts to connect migration with climate change adaptation, disaster risk reduction, resilience, and development [11].

While there is an increasing body of research and analysis focused on climate change-related migration, the impacts on human health are under-examined. Herein, we explore the nexus between climate change, migration, and human health using case studies to examine a range of climatic push factors, diverse migration pathways, and resultant health impacts.

## Health risks

Climate change affects human health. ‘Direct’ or ‘primary’ health impacts include heat-related morbidity and mortality and disaster-related injury. ‘Indirect’ health effects are mediated via changes to ecological and social systems, including, for example, altered fresh water availability and food security, changing disease ecologies including altered distribution of disease vectors and pathogens, and – as is the focus of this article – the health impacts of climate-related migration, displacement, and relocation [12].

**Fig. 1:**
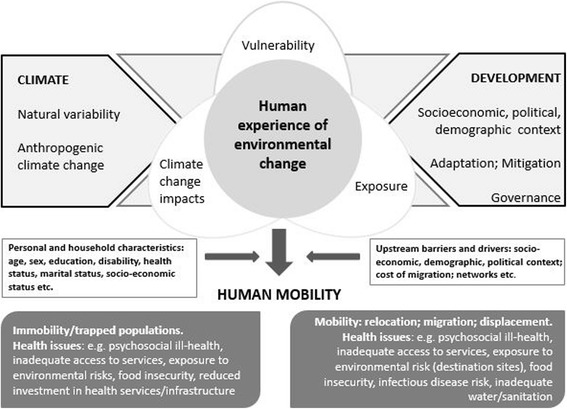
Conceptual framework showing human experience of environmental change as a ‘driver’ of migration and its potential impacts on health (Adapted from [4, 13])

Climate-related migration can potentially have both positive and negative health effects and well-being consequences for both the host and home communities. We have adapted the Intergovernmental Panel on Climate Change risks framework (Fig. 1), using a migration lens, to outline various factors that determine and mediate environmental risks and contribute to migration responses, which culminate in diverse consequences for population health. Climate and weather events, vulnerability, and exposure are influenced by a range of factors, including natural climate variability, anthropogenic climate change, and socioeconomic development. Adaptation to climate change focuses on reducing vulnerability and exposure, and building capacity and resilience to the potential adverse impacts of climate extremes, acknowledging that risks cannot be completely eliminated [13]. Diverse migratory responses, mediated by personal and household characteristics as well as broader drivers and constraints, include immobility, forced displacement, migration, and planned resettlement.

Due to the complexity of ecosystems, and the diversity of place-based impacts, including factors such as governance, socioeconomic status and other societal aspects, it is challenging to draw global conclusions about the influence that climate change exerts on human populations. Therefore, we explore individual case studies where environmental and climatic change is influencing migration and health, and discuss the associated vulnerabilities and resilience. The following case studies cover planned relocation, immobility and in situ adaptation, planned return, rural–urban migration, and circular mobility in a range of diverse settings worldwide.

### Planned relocation in Papua New Guinea

Planned relocation occurs when populations are moved in a preemptive and coordinated way. Planned relocation can be a disaster risk reduction intervention and a form of climate change adaptation. UNHCR stipulates that planned relocation should always be a last resort, based on consent, and should enhance the living standards of relocated communities [14]. Important limitations of planned relocation include its high cost and that it can potentially deplete the economic, human and social capital of both the hosts and relocated populations [15]. Key factors influencing the success of planned relocation include support from host communities, the creation of livelihoods and land security [16].

In the Pacific Island countries, the climate-sensitive health risks of highest priority include trauma from extreme weather events, psychosocial ill-health and factors relating to mental health issues such as population pressures and health system deficiencies [17]. Several Pacific Island countries, including Fiji, Vanuatu, the Solomon Islands, and Papua New Guinea, are planning for and implementing planned relocation in response to sea-level rise. Yet, the associated health risks and opportunities of relocation have received limited consideration.

The independent state of Papua New Guinea, with a population of over 6.1 million [18], has seen the lives and livelihoods of the atoll communities being affected by both slow onset and sudden climatic changes [3]. In north-eastern Bougainville, residents of the Carteret Islands face sea level rise, inundation, soil salinization and land loss. Despite the construction of sea walls and mangrove planting, more than 50% of their land has eroded since 1994. In addition to climate-related sea-level rise, dynamite fishing has weakened the coral reefs, destroying natural barriers, mangrove clearing has contributed to coastal erosion, and tectonic change and cyclical events have contributed to relative sea-level rise [15]. In 2007, the Council of Elders of the Carteret Islands formed an NGO called *Tulele Peisa* (Sailing the Waves on Our Own). Tulele Peisa developed the Carteret Integrated Relocation Project, a community-led relocation model, to coordinate the voluntary relocation of Carteret Islanders to Bougainville Island, 100 km to the north-east. The location of the relocation site was critical to ensure sufficient land for the Carteret families to be economically self-sufficient. The relocation also prioritised food security by ensuring access to traditional fishing grounds, which was important for nutritional and cultural reasons. Additionally, the Carteret Integrated Relocation Project sought to ensure that host communities would benefit from the relocation through an upgrading of health facilities and schools [19]. Nevertheless, despite the apparent opportunity for livelihoods, food security and access to health services, the future through relocation was seen as uncertain and few wished to relocate to the new site.

### Immobility and in situ adaptation in Alaska

For many communities exposed to climate change impacts, migration is not possible. Thus, there is a growing focus on ‘trapped populations’ who have limited social and economic capital and are unable to migrate in the face of climate change threats [4]. Additionally, there are also populations that experience the adverse impacts of climatic change and for whom migration is an assumed adaptive response, but yet choose not to migrate or turn to other in situ adaption options in order to moderate harm [13].

The Northwest Arctic region is home to 12 communities, the largest of which is the Kotzebue, with a population of 8000 residents, 85% of which are Iñupiat. By 2006, the average annual temperature in this isolated region had increased by 1.8 °C relative to pre-industrial levels [20], with such warming manifesting in unusual weather and environmental conditions, including storm surges, coastal erosion, thawing permafrost, delayed freeze-up and earlier ice breakup. While Iñupiat communities anticipate resettlement as a necessary response in coming years, for now they remain in situ due to their sense of attachment to place and lack of government support.

Immobile Iñupiat community members face climate-related population health impacts, including increasing prevalence of climate-sensitive infectious disease, such as pneumonia, and skin infections secondary to deteriorating sanitation caused by climate change-related damage to water and sanitation infrastructure [21]. Mental health impacts related to safety and security concerns as well as a sense of loss due to changing sociocultural and environmental conditions have also been reported. Further, health is adversely impacted by climate-related food insecurity and weather-related injury [21]. Permafrost thawing beneath the lakes that provide drinking water threatens water security and affects agriculture, thus increasing the likelihood of new infectious disease being introduced through harvested and imported foods as well as by vectors migrating northward due to vegetation changes, e.g. beavers harbouring giardiasis [22].

In situ adaptation activities to reduce vulnerability to climate change impacts and health risks include the use of satellite technology to reduce personal injury risk, improved water, sanitation and hygiene facilities, and infrastructure projects such as the construction of revetment walls to inhibit the impact of sea level rise. Further adaptation activities include strengthening disease surveillance and food and water quality monitoring systems [21].

### Forced displacement and planned return in South Sudan

Planned return, also referred to as assisted return, involves financial, logistic and administrative re-integration support to displaced populations returning home. Planned return often involves rejected asylum seekers or stranded migrants who are unwilling or unable to remain in the host country and thus return to their countries of origin [23].

After two decades of civil war in Sudan, a peace treaty was signed in 2005 and, in 2011, the world’s newest nation, South Sudan, was born. However, the new nation was already a failing state with increasing inflation, significant food insecurity and a high proportion of the population reliant on food aid. The country exists on the edge of climate extremes, where climate change acts as a threat multiplier of the very high existing burden of disease coupled with the weak/collapsed health system. Further, climate change significantly impacts livelihoods and health due to the country’s dependence on rain-fed agriculture. With temperatures reaching 50 °C, a minor additional temperature increase may trigger crop failure, amplifying significant existing food insecurity. As malnutrition underlies a large proportion of morbidity and mortality, health would thus be negatively affected in all demographic groups, especially children. Additionally, climate change will also amplify the existing high burden of climate-sensitive disease, including malaria, leishmaniasis and diarrhoeal diseases [24, 25]. In the broader context, Africa holds the largest global share of poverty, the highest rate of undernourishment, and is projected to have the highest population growth and above-average climate change, making the region particularly vulnerable to migration and its associated health impacts [26].

Conflict and economic downturn have contributed to decimated livelihoods through livestock loss due to disease and theft, crop destruction and delayed planting due to displacement during conflict [24]. More than 2.3 million people, or one in five people, in South Sudan have been forcibly displaced since the most recent conflict began in 2013. This includes 1.6 million internally displaced people, of whom 50% are children, and nearly 646,000 refugees in neighbouring countries [24]. Displacement in South Sudan is due to multiple interconnected threats, including climatic shocks, inter-communal violence and economic decline [24]. There is also a high degree of secondary displacement due to food and water shortages, lack of education and healthcare services, and few job opportunities in receiving areas.

Planned relocation was facilitated in some South Sudanese regions by identifying areas with high returnee loads and undertaking comprehensive village assessments of basic services and resources in receiving areas in order to reduce the risk of secondary displacement, improve the capacity of receiving communities, and promote sustainable planned return [23]. Importantly, the assessments were coducted in contexts with high levels of exposure and vulnerability to climate change impacts and associated adverse health outcomes. The village assessments included a comprehensive account of access to basic services and resources in receiving areas and were used to plan and prioritise capacity-building programmes that would benefit both host and returnee populations. Further, risk factors that might contribute to conflict over scarce resources were identified and mitigated, supporting the peace-building process [23].

### Rural–urban migration in Northern Australia

Rural to urban migration involves temporary or permanent population movement from rural settings to urban centres. In addition to existing global rural–urban migration flows, climate change is expected to increase this migration pathway in many climate vulnerable regions [3].

‘Northern Australia’, including parts of Queensland, the Northern Territory and Western Australia, is home to an indigenous population of 160,000 people. Natural hazards that are projected to be exacerbated by climate change in this region include cyclones and storm surges, flash floods, heat waves, coastal erosion, bushfires and drought [18]. Indigenous communities in the region have a high capacity to adapt to climate change impacts due to their long history of building environmental knowledge and connection to country [27]. However, other studies of indigenous populations elsewhere indicate complex climate–migration mental health impacts on both the host and migrant communities due to the disruption of social ties, with deleterious implications for those left behind as well as those on the move [28].

Some indigenous communities in rural and remote Northern Australia have temporarily migrated to urban areas in response to environmental changes, including wet season migration from out-stations to avoid being cut-off due to flooding [29]. Other reasons for rural to urban migration include health, livelihood and employment opportunities, and the need to be closer to essential services following the centralisation of many government services. Besides urban migration, alternative adaptations pursued by indigenous people, government agencies and community groups include replacing and relocating infrastructure, strengthening food and water availability, improved health preparedness, and reorganising work, social and cultural schedules [30].

There is extensive evidence that transitions to sedentary, urban and western lifestyles in indigenous communities have led to the increased prevalence of non-communicable diseases, such as obesity, cardiovascular disease and type 2 diabetes, as well as mental health problems related to social change and substance abuse [31]. If climate change-related mobility amplifies these transitions, including via urbanisation, it might further undermine the health and wellbeing of indigenous peoples. Nonetheless, the evidence to date on indigenous climate-related migration indicates great diversity in terms of exposure to impacts and capacity to respond; therefore, generalised conclusions about migration as adaptation are unlikely to emerge [32, 33].

### Temporary and circular mobility in Colombia and Spain

Circular mobility is a temporary form of migration where the migrant worker, family or community moves between home and host areas typically for the purposes of employment [34]. It is broadly considered as positive since it can benefit the home population through remittances and the host population through the provision of skills and labour. Further, migration experts argue that the individual and structural benefits of labour mobility outweigh concerns about ‘brain drain’ [35]. However, circular migration may have negative implications, including cultural change, social exclusion, family fragmentation and high levels of dependency on the mobile individual.

Colombia is considered highly vulnerable to the negative impacts of climate change despite contributing only 0.2% to the total global CO_2_ emissions [36]. There has been a temperature increase of 1 °C in the last two decades; further, the temperature increase in the Andes was 70% greater than the average global temperature change [35]. More frequent La Niña events, wetter than average conditions resulting in flooding, and an increase in extreme weather events are currently experienced in the region [23, 37]. Dengue fever, malaria, leishmaniasis and leptospirosis are climate-sensitive diseases of primary concern in the country [38]. Additionally, the prevalence of acute respiratory infections has increased and is related to intensified rainfall, changes in ambient temperature and the circulation of respiratory viruses [37].

The Colombian Temporary and Circular Labour Migration programme, an innovative model of labour migration between Spain and Colombia established in 2009, involved over 1500 people who received access to financial services and technical training to enable temporary migration. The programme provided temporary work abroad for families affected by natural disasters and prevented anticipated rural to urban slum migration, allowing affected areas to recuperate from slow- and fast-onset climatic shocks. People who temporarily migrated were also able to provide support to communities at home through remittances, which served to aid the recovery of affected areas. This international cooperation prioritised the most vulnerable populations and rural communities and offered leadership and local development training to women, potentially contributing to the long-term sustainable development in the region [15].

## Response strategies

The case studies above illustrate diverse migration responses that communities and governments employ in retort to environmental change. These migration responses can occur both within and between countries and can have differential impacts on health and well-being. While it is not possible to definitively state that the issues delineated in each case study are directly attributable to climate change, it is accepted that many environmental risks will increase in frequency and intensity due to climate change. Notwithstanding this uncertainty, it is important for researchers, practitioners and policy-makers to further consider the manner in which communities might migrate (e.g. proactively or reactively, forced or voluntarily) in response to current and/or future changes in their environment. Uncertainty should not imply inaction; rather, public health researchers and practitioners should support the development of appropriate and well-considered responses with multiple benefits to communities, regardless of the degree of climate migration that might be experienced.

International initiatives have developed principles, frameworks and statements around climate-related migration, including the Cancún Adaptation Framework [38], which recognised climate change-induced displacement, migration and planned relocation as central to climate change adaptation. Yet, multi-sectoral and multi-agency involvement in climate change decision-making and broader governance approaches is complex [39]. Climate migration will require active responses from intergovernmental organisations, all tiers of government in nation states, as well as partnerships beyond government, including NGOs and the private sector. This could include partnership webs involving the International Organization for Migration, UNHCR, Ministries of Health, Departments of Environment, and humanitarian aid organisations like Medecins Sans Frontieres and the International Committee of the Red Cross.

Given the difficulty of attributing migration to climate change impacts, it is critical to develop and support migration-related frameworks and focus broadly on the intersections between migration, development and environmental change [40]. The WHO Framework for Climate Resilience provides programmatic direction for strengthening health systems by improving universal health coverage and ensuring services are migrant inclusive and responsive to migration flows. This provides an important foundation for government policy and programmes, providing guidance on how to link health systems strengthening with climate change resilience. Support with the operationalisation of this framework is an important next step.

Climate-related migration, as with any migration, can contribute to both vulnerability and resilience [23, 41], with potential for both protective and detrimental impacts on human health and well-being. For example, migrating populations may be exposed to infectious diseases for which they have limited immunity or face new climate-related health risks in the destination context; yet, they may also migrate from sites of food insecurity or have improved access to health services in destination sites. Destination contexts, including political and legal frameworks, physical infrastructure, education and employment opportunities, technology and social capital, will operate as key determinants of migrant health [42].

Climate-related migration will often be a last resort. Vulnerable communities must be supported to better prepare for and respond to climate change risks, recognising that many communities have substantial experience in planning for and recovering from natural hazards. Yet, when people choose to migrate, relocate or are forcibly displaced, it is critical to reduce the health risks and foster opportunities for the improved health and well-being of people on the move.

## Conclusion

Herein, we discussed diverse migration experiences and the associated health outcomes as analogues to explore the climate change–migration–health nexus. These are illustrative examples rather than predictive cases since migration experiences and outcomes are diverse and context based. Thus, forecasting requires causal thinking that winds its way through uncertain climatic, environmental, geomorphological and population health contexts.

A key fact for this emerging issue is the need to strengthen health systems to make them both more climate resilient and migrant inclusive. A shift to ‘climate-resilient health systems’ is a useful precautionary measure, as it aims to strengthen multiple aspects of national and sub-national health systems, regardless of the extent to which climate-related migration might occur.

Further, there is a need for empirical evidence of the health impacts of climate-related migration, including for migrating populations and host communities, as well as populations left behind. While there is emerging research that examines how migration can be an adaptive response to climate change, the health impacts – both positive and adverse – must be better understood. The central question is whether people, communities and nations can adopt migration as a response to climate change impacts, whilst safeguarding human health and well-being. This requires a better understanding of risk of infectious diseases, non-communicable diseases, food security, environmental exposures such as heat extremes, mental health and access to health services.

Migration due to changes in natural environments is complex. The adaptive potential of migration cannot be assumed, particularly as migration itself can induce health risks. Rights-based health and governance frameworks are required such that migrating communities have the opportunity to build healthy lives in sites of resettlement or relocation. We should strive for research, science, governance and policy that contributes to healthy futures and synergises with the broader development agenda.
